# The effect of listening to preferred music after a stressful task on performance and psychophysiological responses in collegiate golfers

**DOI:** 10.7717/peerj.13557

**Published:** 2022-06-01

**Authors:** Hung-Tsung Wang, Yung-Sheng Chen, Ghazi Rekik, Chia-Chen Yang, Mao-Sheng Lai, Hsia-Ling Tai

**Affiliations:** 1 Graduate Institute of Sports Training, University of Taipei, Taipei, Taiwan; 2 Department of Exercise and Health Sciences, University of Taipei, Taipei, Taiwan; 3 Tanyu Research Laboratory, Taipei, Taiwan; 4 Research Laboratory: Education, Motricity, Sport and Health (LR19JS01), High Institute of Sport and Physical Education, Sfax University, Sfax, Tunisia; 5 Department of Leisure and Recreation Management, Taipei City University of Science and Technology, Taipei, Taiwan; 6 Department and Graduate Institute of Physical Education, University of Taipei, Taipei, Taiwan

**Keywords:** Psychological stress, Golf performance, Music intervention, Autonomic nervous system

## Abstract

**Background:**

This study explores whether listening to preferred music after a stressful situation affects putting and swinging performance, heart rate (HR), HR variability (HRV), and anxiety among amateur golfers.

**Methods:**

Twenty healthy amateur collegiate golfers voluntarily participated in this study (age 20.1 ± 1.17 yrs., height = 173.8 ± 7.74 cm, body weight = 72.35 ± 12.67 kg). Pre- and post-intervention HR and HRV measurements were taken, along with a self-report of the State-Trait Anxiety Inventory (STAI-S) and Triple Factor Anxiety Inventory (TFAI). Participants were exposed to a stressful situation through the Stroop Colour and Word Test (SCWT) and then instructed to perform three golf-practice sessions in a golf simulator, separated by 48–72 hours of recovery, under different conditions: control, pre-task music, and synchronised music.

**Results:**

No significant difference was identified between the experimental conditions for swinging (in terms of total distance (*p* = 0.116), carry distance (*p* = 0.608), speed of the ball (*p* = 0.819), and launch angle (*p* = 0.550) and putting performance (the number of successful putts on target (*p* > 0.05) and distance error between the target and ball (*p* = 0.122). No main effect for condition and time of intervention, as well as no interaction between these two factors was found for HR, HRV, and STAI-S (*p* = 0.116). However, the pre and post-intervention percentages of physiological items of the TFAI indicated a large, significant difference in synchronised music trial (*p* = 0.012, pre-task trial = −1.92% < control trial = 0% < synchronised trial = 4.58%).

**Conclusions:**

The results imply that following a stressful situation, listening to preferred music before and/or during golf has no immediate effect on golf performance, anxiety, and psychophysiological responses in collegiate golfers.

## Introduction

Improving performances and self-motivational beliefs constitutes one of the major issues in the sports-exercises domain (Belkhir et al., 2020). However, mental stress and/or fatigue may exert disruption on task performance as well as other psychological (*e.g.*, anxiety) and physiological (*e.g.*, heart rate (HR)) responses among athletes (*e.g.*, Cooke et al., 2011; Badin et al., 2016; Van Cutsem et al., 2017; Filipas et al., 2021; Habay et al., 2021). For example, it was found that pressured situations could decrease HR variability (HRV), which can reflect increased mental effort or increased HR, which can reflect an increase in anxiety and/or arousal (Veldhuijzen van Zanten et al., 2002). In the same vein, Cooke et al. (2011) suggested that elevated competitive pressure elicits disruption of golf performance (*i.e.,* putting accuracy) through both psychological (*i.e.,* anxiety, effort) and physiological (*i.e.,* HR) pathways. Moreover, mental fatigue is associated with impaired physical activity, and technical and tactical performances during small-sided games (*e.g.*, Badin et al., 2016; Trecroci et al., 2020). Furthermore, a recent systematic review (Habay et al., 2021) revealed that mental fatigue could also impair a myriad of sport-specific psychomotor performances, including decision-making, reaction time, and accuracy outcomes.

The current state of literature indicates that listening to music is a valuable strategy for improving task performance and certain psychological, physiological, or psychophysical responses (Belkhir et al., 2022). Due to its ability to act as a stimulant and/or relaxant (Bishop, Karageorghis & Loizou, 2007), this auditory stimulus can promote greater athletic performance in different exercise settings (*e.g.*, Simpson & Karageorghis, 2006; Belkhir et al., 2019; Khemila et al., 2021). Moreover, listening to music is usually associated with better psychological states by increasing the level of motivation/enjoyment and counteracting the negative dimensions of mood such as anxiety and tension (Stork, Karageorghis & Ginis, 2019; Belkhir et al., 2020). Additionally, listening to music could guarantee better psychophysical states by reducing the perceived effort and fatigue (Yamashita et al., 2006). Furthermore, listening to music can have positive effects on physiological (*e.g.*, HR; Thakare, Mehrotra & Singh, 2017) and biochemical parameters (*e.g.*, blood lactate concentration; Eliakim et al., 2012). These presumed advantages of music intervention have been reported in different sports activities, such as swimming (*e.g.*, Karageorghis et al., 2013), running (*e.g.*, Chtourou et al., 2012; Terry et al., 2012), cycling (*e.g.*, Bigliassi et al., 2017), soccer (*e.g.*, Belkhir et al., 2019), and volleyball (*e.g.*, Eliakim et al., 2007).

The added value of listening to music is also attested in past scientific works within the sport of golf. Golf is a closed kinetic chain sport that requires fine and gross motor control skills. To achieve a successful golf shot (*i.e.,* hit the ball close to the target), practitioners should succeed in both “*swinging*” and “*putting*” performances. The full swing is a complex dynamic movement that requires excellent coordination of different body segments (*e.g.*, head, upper/lower limbs, and trunk), and control of the velocity of swing performance during the golf swing cycle (Sheehan, Watsford & Pickering Rodriguez, 2019). The putting stroke is a sophisticated action that requires stable motor performance and mental status for the accuracy of shots (Richardson, Mitchell & Hughes, 2018). Baghurst et al. (2014) showed that listening to music (classical, country, rock, jazz, and hip hop/rap) during exercise improved golf-putting performance, where jazz was the most effective music type. Another qualitative study explored self-reported music use among amateur and semi-professional golfers (Gabana et al., 2019). The results demonstrated that the use of music could help golfers to maintain attention, achieve optimal physiological arousal (*e.g.*, through increased energy or calm nerves), regulate their mood (particularly following a poor shot or round), and improve psychological states (*e.g.*, confidence, motivation, adherence, flow, and enjoyment). More recently, Wang et al. (2020) investigated the effects of listening to preferred music on the golf swing and putting performance, HR, HRV, and anxiety in collegiate golfers. The authors demonstrated that listening to self-selected music, either before or during exercise, could guarantee better cardiac-related responses and decrease anxiety levels.

The current study was designed to extend the research base by examining the relationships between music and sports performance. More specifically, it explored whether listening to preferred music (before and during exercise) following mental stress/fatigue would affect golf performance (*i.e.,* putting and swinging), HR, HRV, and anxiety in amateur golfers. To the best of our knowledge, no previous research has addressed this issue in sports (in general) and golf (in particular), which is a significant contribution of the current study. Based on the literature, it was hypothesised that listening to preferred music following mental stress/fatigue would improve swinging and putting performance, and would result in better psychophysiological responses in collegiate golfers.

## Materials & Methods

### Experimental approach to the problem

**Figure 1:**
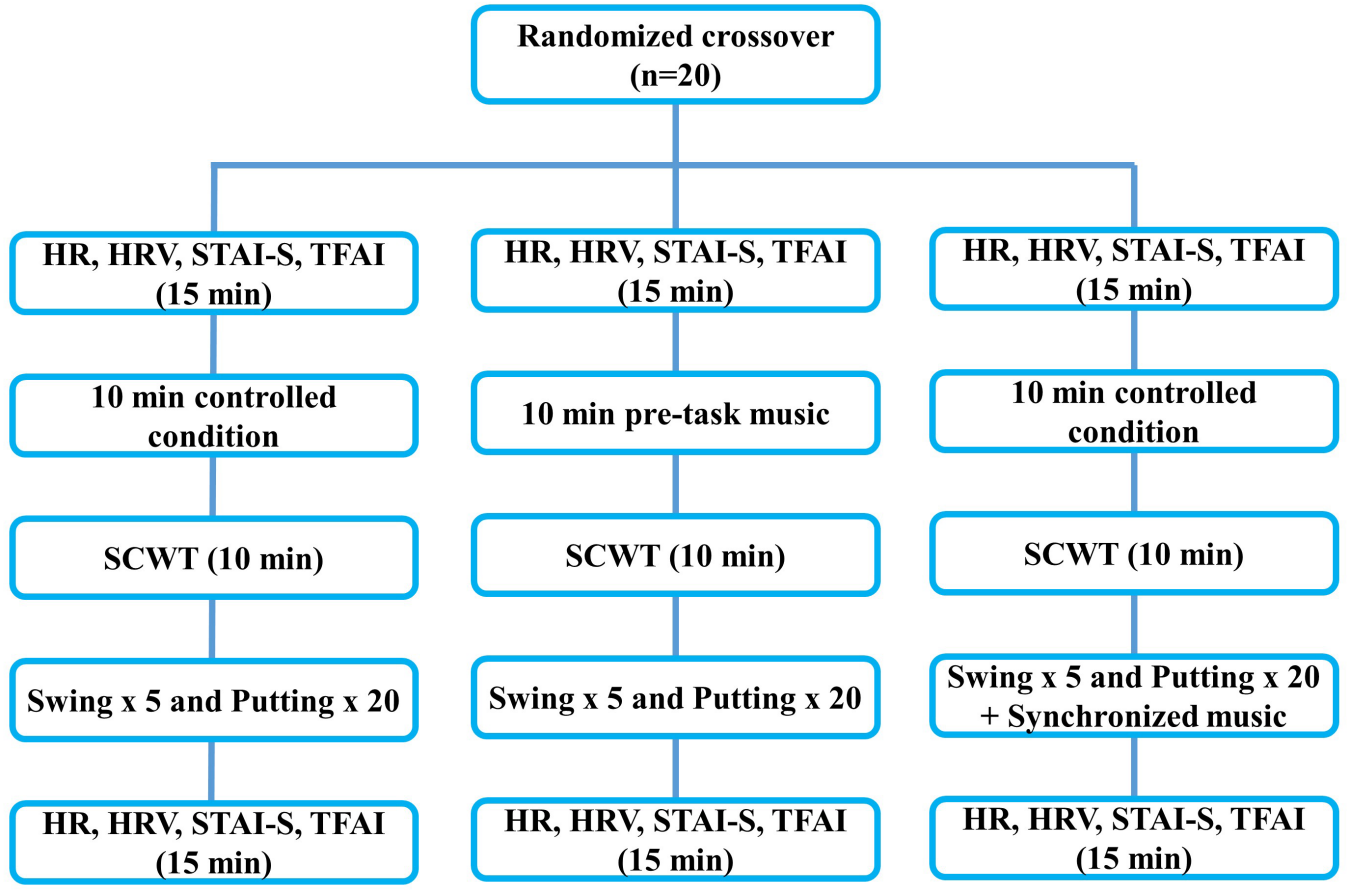
Flow diagram of the study experiment. HR, heart rate; HRV, heart rate variabiltiy; STAI-S, state-trait anxiety inventory; TFAI, triple-dimension anxiety; SCWT, Stroop Color and Word Test; Min, minutes.

A counterbalanced measure and within-subjects design was used in this study. This laboratory-based experiment investigated the effects of pre-task and synchronised self-selected music interventions on golf performance, cardiac-related responses, and anxiety in collegiate golfers. The participants were a part of the control trial, pre-task music trial, and synchronised music trial in a randomised order, 48–72 hrs apart (Fig. 1). A web-based program (https://www.randomizer.org/) was used for randomisation. The golf swing and putting performances were evaluated in a virtual golf simulation environment. Additionally, baseline and post-performance measurements of cardiac-related responses and anxiety status were taken.

### Participants

Sixteen male and four female collegiate golfers at the amateur level (age 20.1 ± 1.17 yrs., height = 173.8 ± 7.74 cm, body weight = 72.35 ± 12.67 kg) voluntarily participated in this study*.* All participants were healthy with no contraindication to participation. The inclusion criteria were as follows: (1) Collegiate golfers and (2) a training frequency of 4–5 times a week (weekly training time of 10–12 hrs). The exclusion criteria were as follows: (1) history of severe neuromuscular injury, (2) lower extremity or lower back injuries within six months, and (3) current diagnosis of cardiovascular or metabolic diseases. All participants signed informed consent forms and were familiarised with the experimental procedures one week before the experiment. Ethical approval was granted by the Institute Ethics Committee of the University of Taipei (UT-IRB-2021-010). This study was performed per the ethical standards of the Institutional Human Ethical Committee of the Declaration of Helsinki and its later amendments.

The sample size was determined based on our recent study (Wang et al., 2020), using a setting of *a priori* compute required sample size: analysis of variance (ANOVA) repeated measures within factors and an *α* level of 0.05 (G*Power 3.1.9.4, Düsseldorf, Germany). The results revealed that 20 participants approached an actual power (1–*β* error probability) of 0.81.

### Experimental procedure

**Figure 2:**
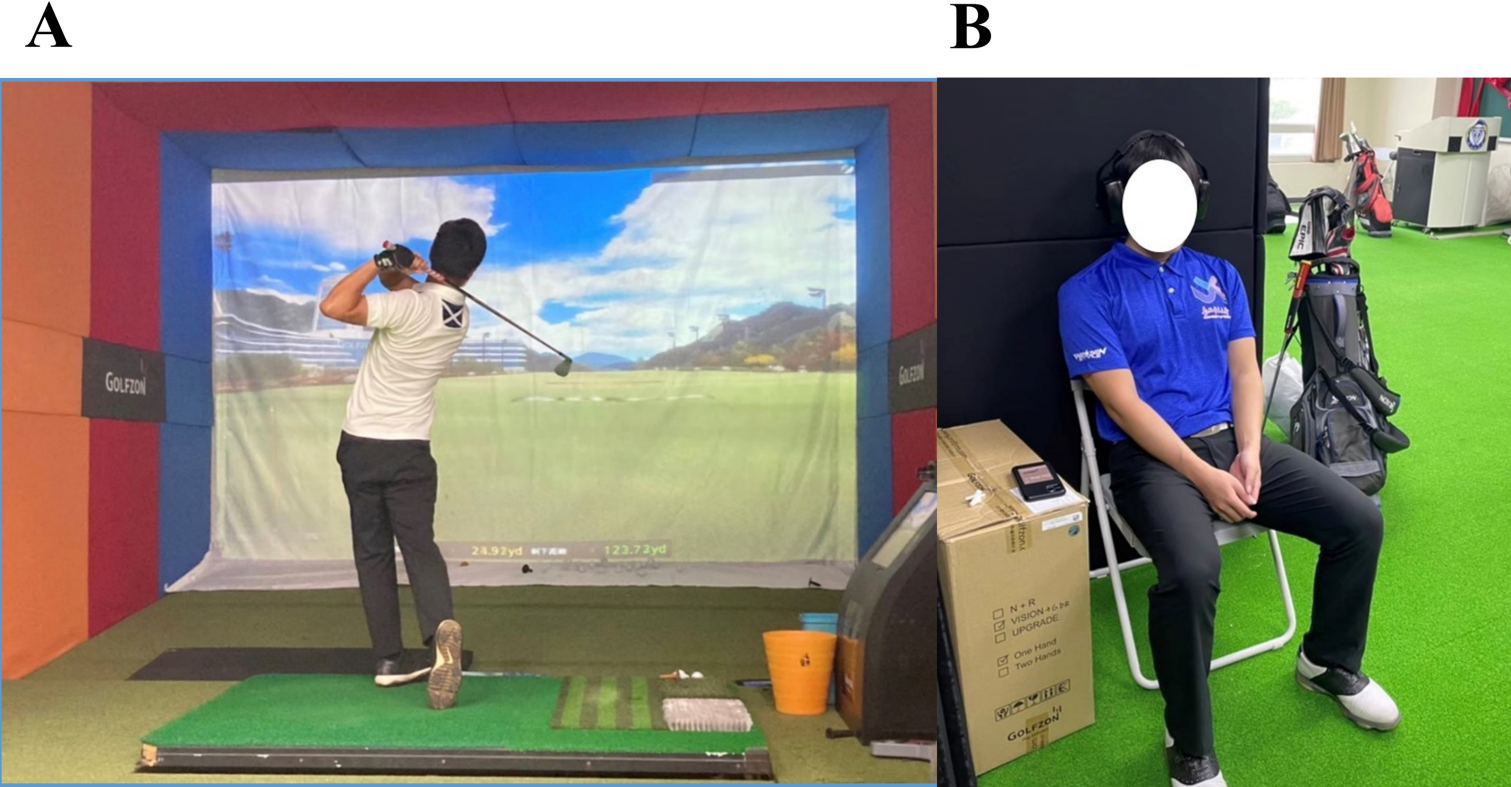
Experimental setting for assessment of golf performance (A) and music intervention (B).

Participants who met the inclusion and exclusion criteria first visited the laboratory for familiarisation and determination of individual preference in music and physical characteristics. Afterwards, the participants visited the laboratory on three separate occasions for control, pre-task music, and synchronised music trials with a 48–72 h interval between each trial. For each trial, a baseline measurement of resting HR and HRV was taken for 10 min in a seated position. The participants were then requested to answer the State-Trait Anxiety Inventory (STAI-S) and Three-Factor Anxiety Inventory (TFAI). Subsequently, the participants were exposed to a 10-min long pre-task music intervention or control condition (resting in a seated position for 10 min), followed by a 10 min Stroop Colour and Word Test (SCWT). During the assessment of golf performance, the participants performed five swings and twenty putts in a virtual golf environment (Fig. 2). During the synchronised music trial, the participants carried out their swings and putts while listening to the self-selected music. Finally, HR, HRV, STAI-S, and TFAI were assessed again during the post-exercise measurement. All trials were conducted at the same time of day.

### Music intervention

Personal preference in music was used as 15 min long music intervention. All participants selected pop music as their preferred music. The participants were instructed to sit on a comfortable chair and use their smartphones to play music *via* personal earphones. To prevent background noise a 3M protective earmuff (noise reduction rating of 27 dB, 1427, 3M, China) was used while listening to music. The music was defined as slow tempo music (<120 bpm) or fast tempo music (>120 bpm), per a study by Karageorghis, Terry & Lane (1999). BPM analyser software was used to analyse the self-selected music tempo (Mixmeister, Cumberland, RI, USA).

### Mental stress

A Chinese version of the SCWT was used to induce mental stress before the golf performance. The SCWT consisted of two subtasks: (1) naming Chinese colour words that were printed in the same colour as the word (simple identification) and (2) naming the colour of Chinese colour words printed in a different colour. The colours of the characters were black, blue, red, and yellow. The participants were asked to name the colours from the two subtasks left-to-right and top-to-bottom. They were then asked to reverse the naming order, *i.e.,* from right-to-left, top-to-bottom, and repeat the entire process as quickly as possible in 10 min. The authors have permission to use this instrument from the copyright holders (Wang et al., 2018).

### Golf performance assessments

An indoor golf simulation system (Vision Compact, Golfzon, Korea) was used to assess golf performance. It was set to the driving range mode for swing performance, and the challenge mode, with a putting distance of 2.5 yards, for putting performance. Each participant used their seven-iron and putter and was asked to swing five times and putt twenty times. The performance of each shot was displayed on the screen using a Golfzon GDR automatic detector. The parameters of swing performance included flight distance with carry and roll distance (flight), flight distance, speed of the ball (speed), and launch angle (angle), while the parameters of putting performance included the number of successful trials (putting performance) and the error distance between the hole and ball in unsuccessful trials (putting distance). All participants were familiarised with golf practice in the Golfzon virtual environment during their routine training sessions.

### Cardiac-related response

Heart rate and HRV were used to evaluate autonomic nervous adaptation in response to music intervention. A portable HR monitor (Polar RS800CX, Polar Electro, Kempele, Finland) was used to record resting cardiac-related responses while seated for 10 min.

The record of the first 5 min was not included in the analysis of HR and HRV indices to avoid the impact of orthostatic effects on autonomic nervous activity. All raw data were processed using professional HRV analysis software (Premium version 3.2.0, Kubios, Kuopio, Finland). A prior setting of moderate artefact correction and smoothing at 500 Lambda were used to process the data. The HRV indices included (1) time-domain analysis: standard deviation of normal R-R interval (SDNN) and mean sum of the squared differences between RR (RMSSD); (2) frequency domain analysis: low-frequency power spectrum (LF) and high-frequency power spectrum (HF); and (3) nonlinear analysis: standard deviation of the points perpendicular to the line of identity (SD1) and standard deviation along the line of identity (SD2). The spectrum of frequency bands for the LF and HF were set to 0.04–0.15 Hz and 0.15–0.4 Hz, respectively.

### Anxiety assessment

Two Chinese versions of leading anxiety measures were used to evaluate anxiety in this study: the STAI-S and TFAI.

The STAI-S consists of 20 items related to individual awareness of anxiety (Spielberger, Gorsuch & Lushene, 1970), presented as a four-point Likert scale ranging from one (fully disagree) to four (fully agree). The sum of the scores for 20 items was used for the statistical analysis. The minimum STAI-S score was 20 points, while the maximum STAI-S score was 80 points. The development of the STAI-S scores was positively correlated with anxiety levels. The Cronbach’s alpha coefficients of the Chinese version of STAI-S were 0.90 and 0.81 for the State and Trait scales, respectively (Shek, 1988). The authors have permission to use this instrument from the copyright holders (Shek, 1988).

The TFAI is a useful tool for quantifying the various psychological factors for anxiety and depression during exercise (Cheng, Hardy & Woodman, 2011). Thus, we used this tool to evaluate the impact of anxiety levels on golf performance. The Chinese version of the TFAI consists of 21 items, divided into three components. Items 1–10 evaluate cognitive components (four items for anxiety and six items for self-awareness), 11–17 evaluate physiological components (four items for autonomic nervous responses and three items for somatosensory feedback), and 18–21 evaluate perceived components. A five-point Likert scale was used to quantify psychological feedback (from 1 = fully disagree to 5 = fully agree). The internal consistency of Cronbach’s alpha ranged from 0.85 to 0.86 (Cheng, Hardy & Woodman, 2011). The authors have permission to use this instrument from the copyright holders (Cheng, Hardy & Woodman, 2011).

### Statistical analyses

Descriptive data of the measured variables were presented as mean and standard deviations (mean ± SD) or median (interquartile range [IQR]). The normal distribution of the study variables was evaluated using the Shapiro–Wilk test. A repeated measure ANOVA was used to compare putting performance, STAI-S, HR, and HRV. When a significant interaction or main effect was identified, a post-hoc analysis with Bonferroni contrast was used to identify the difference between the mean values. As a significant level of normality was identified in swinging and TFAI, nonparametric tests were performed for subsequent comparisons. The Friedman test with the Monte Carlo adjustment was performed to compare experimental conditions. The Wilcoxon rank test was used to identify differences between the baseline and post-intervention pairwise comparisons. The partial eta squared (*η*_p_^2^) was used for all repeated measure comparisons of effect size (Cohen, 1988). Additionally, the percentage change in STAI-S and TFAI at baseline and post-intervention assessment was calculated as

$\text{%}variable=( \frac{Variabl{e}_{post}-Variabl{e}_{baseline}}{Variabl{e}_{baseline}} )\times 100$.

An alpha value of *p* < 0.05 was set for significant differences between means or medians. All statistical analyses were performed using SPSS version 25.0 software for Windows (IBM, Armonk, NY, USA).

## Results

### Physical characteristics and golf level

The physical characteristics and golf levels of the participants are listed in Table 1. Golf performance ranged from par +2 to +13.

**Table 1:** Physical characteristics and competitive level of the participants.

Number	Gender	Age (yrs)	Height (cm)	Weight (kg)	Handicap	Music tempo (bpm)
1	male	22	180	82	5	119, slow tempo
2	male	22	185	80	10	117, slow tempo
3	male	21	169	72	7	128, fast tempo
4	male	21	165	66	7	135, fast tempo
5	male	19	183	69	5	124, fast tempo
6	male	19	183	80	7	148, fast tempo
7	male	19	181	74	7	126, fast tempo
8	male	19	172	68	7	146, fast tempo
9	female	21	163	53	7	130, fast tempo
10	male	18	169	66	6	125, fast tempo
11	female	21	161	53	6	124, fast tempo
12	male	21	180	85	10	103, slow tempo
13	female	20	166	65	2	111, slow tempo
14	male	21	178	62	3	132, fast tempo
15	male	19	173	72	9	99, slow tempo
16	male	19	178	90	3	97, slow tempo
17	male	19	172	65	6	112, slow tempo
18	male	21	178	90	4	116, slow tempo
19	male	20	179	99	6	122, fast tempo
20	female	20	161	56	13	130, fast tempo

**Notes.**

Yrs, years; cm, centremeters; kg, kilogram; bpm, beat per minute. Slow tempo music frequency < 120 bpm; fast tempo music frequency > 120 bpm.

### Golf performance

**Figure 3:**
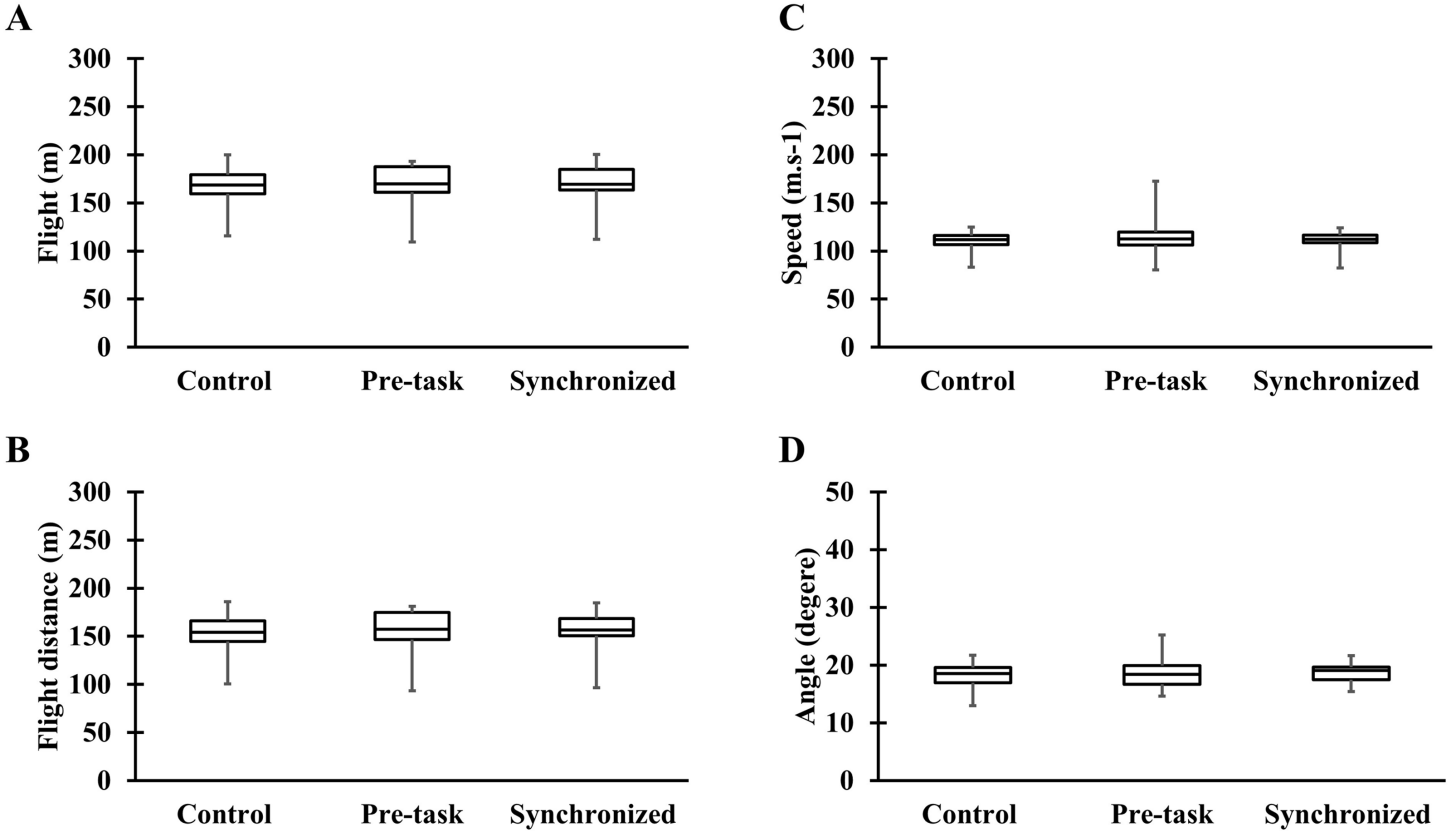
Results of golf swing performance in control, pre-task, and synchronized music trials. (A) Flight distance with carry and roll distance (flight); (B) Flight distance; (C) Speed of the ball (speed); (D) Launch angle (angle).

**Figure 4:**
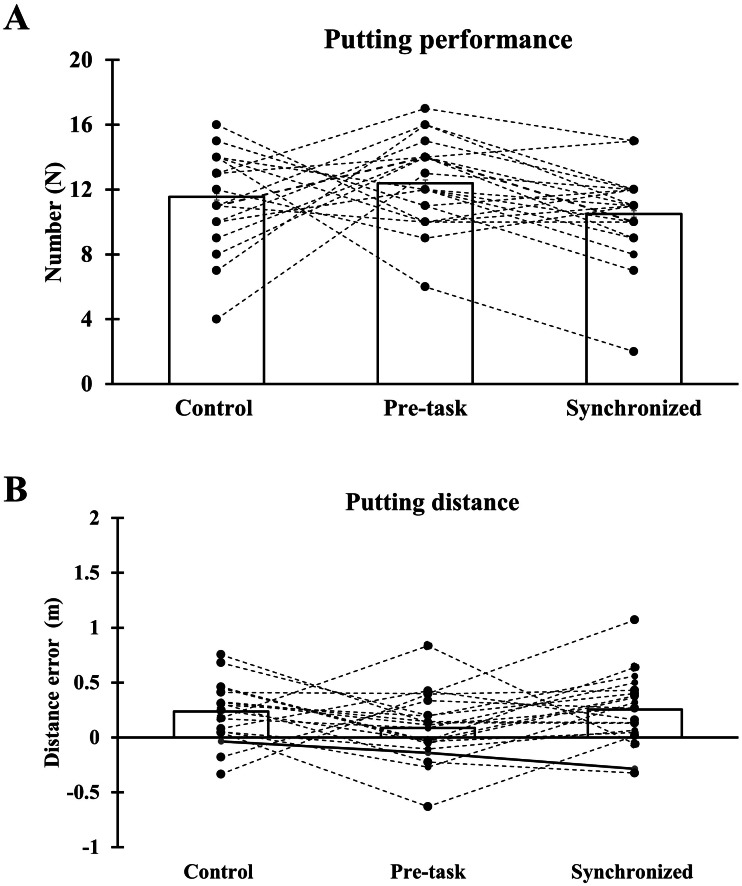
Pooled and individual putting performance data among control, pre-task, and synchronized music trials. (A) Number of successful putting on goal in control, pre-task, and synchronized music conditions. (B) Distance error between hole and ball in unsuccessful performance in control, pre-task, and synchronized music conditions.

Figure 3 demonstrates no significant difference in any golf swing variables (flight (*p* = 0.116), flight distance (*p* = 0.608), ball speed (*p* = 0.819), and angle of ball travelling (*p* = 0.550)) among the experimental trials. Additionally, no significant difference was found in the number of successful putts on target (*F* (2,38) = 2.156, *p* = 0.130, *η*_p_^2^ = 0.102) and distance error between the target and ball (*F* (2,38) = 2.227, *p* = 0.122, *η*_p_^2^ = 0.105) in statistical comparisons (Fig. 4).

### Autonomic nervous function

The results of a two-way repeated-measures ANOVA showed no interaction and main effect of trial or time on mean HR, SDNN, RMSSD, LF, HF, SD1, and SD2 (see Table 2).

**Table 2:** Results of heart rate and heart rate variability parameters during baseline and post-golf measurement in control, pre-task, and synchronized music trials.

Variables	Baseline	Post-golf	Interaction/ Main effect	F ratio	*P*-value	*η*_p_^2^
HR (log)						
Control	4.42 ± 0.15	4.42 ± 0.14	Trial ×Time	0.162	0.851	0.008
Pre-task	4.44 ± 0.10	4.44 ± 0.09	Trial	0.701	0.502	0.036
Synchronised	4.45 ± 0.15	4.45 ± 0.13	Time	0.153	0.700	0.008
SDNN (log)						
Control	3.33 ± 0.54	3.36 ± 0.44	Trial ×Time	0.057	0.945	0.003
Pre-task	3.26 ± 0.39	3.30 ± 0.28	Trial	1.035	0.365	0.052
Synchronised	3.22 ± 0.45	3.23 ± 0.32	Time	0.404	0.533	0.021
RMSSD (log)						
Control	3.06 ± 0.73	3.02 ± 0.54	Trial ×Time	0.652	0.527	0.033
Pre-task	2.93 ± 0.47	2.99 ± 0.32	Trial	0.916	0.409	0.046
Synchronised	2.89 ± 0.59	2.91 ± 0.48	Time	0.030	0.863	0.002
LF (log)						
Control	4.16 ± 0.22	4.27 ± 0.18	Trial ×Time	2.038	0.157	0.097
Pre-task	4.24 ± 0.24	4.22 ± 0.27	Trial	0.116	0.829	0.006
Synchronised	4.19 ± 0.27	4.23 ± 0.23	Time	2.496	0.131	0.116
HF (log)						
Control	3.44 ± 0.51	3.20 ± 0.48	Trial ×Time	1.307	0.283	0.064
Pre-task	3.21 ± 0.60	3.25 ± 0.56	Trial	0.385	0.683	0.020
Synchronised	3.32 ± 0.57	3.21 ± 0.75	Time	2.929	0.103	0.134
SD1 (log)						
Control	2.72 ± 0.73	2.67 ± 0.54	Trial ×Time	0.652	0.527	0.033
Pre-task	2.59 ± 0.47	2.65 ± 0.32	Trial	0.916	0.409	0.046
Synchronised	2.55 ± 0.59	2.56 ± 0.48	Time	0.030	0.864	0.002
SD2 (log)						
Control	3.59 ± 0.51	3.63 ± 0.43	Trial ×Time	0.070	0.933	0.004
Pre-task	3.53 ± 0.39	3.57 ± 0.29	Trial	0.985	0.383	0.049
Synchronised	3.49 ± 0.43	3.50 ± 0.31	Time	0.507	0.485	0.026

**Notes.**

Data are presented as mean and standard deviation. HR, heart rate; log, nature logarithm; SDNN, standard deviation of normal R-R interval; RMSSD, mean sum of the squared differences between RR; LF, low-frequency power spectrum; HF, high-frequency power spectrum; SD1, standard deviation of the points perpendicular to the line of identity; SD2, standard deviation along the line of identity.

### Anxiety status

**Figure 5:**
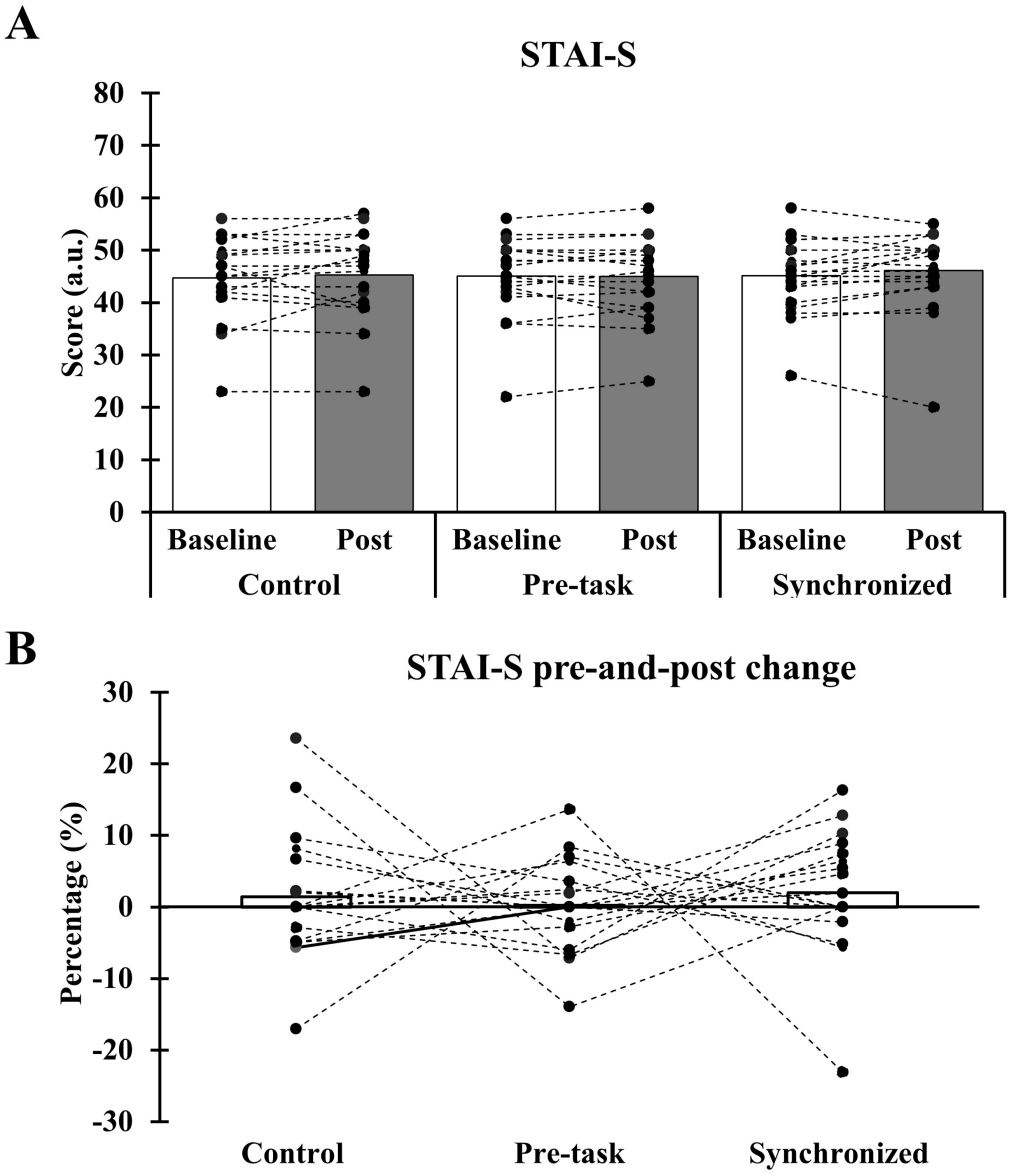
Comparisons of STAI-S during baseline and post-golf measurement in control, pre-task, and synchronized music conditions. (A) STAI-S score during baseline and post-golf test. (B) Percentage change of STAI-S score in control, pre-task, and synchronized music conditions. Pooled and individual STAI-S score among three testing conditions.

The STAI-S results showed no interaction (*F* (2,38) = 0.471, *p* = 0.628, *η*_p_^2^ = 0.024) and main effect (Trial: *F* (2,38) = 0.471, *p* = 0.673, *η*_p_^2^ = 0.021; Time: *F* (2,38) = 3.379, *p* = 0.082, *η*_p_^2^ = 0.151) (Fig. 5A). The pre- and pos *t*-test percentage change of the STAI-S had no main effect of trials (*F* (2,38) = 0.213, *p* = 0.809, *η*_p_^2^ = 0.011) in the ANOVA test (Fig. 5B).

The TFAI results show that the Friedman test (inter-trial comparison) demonstrates a significant difference in the pre- and pos *t*-test percentage of physiological elements (*p* = 0.012, pre-task trial = −1.92% <control trial = 0% <synchronised trial = 4.58%). However, no statistical differences were found in other comparisons (*p* > 0.05) (Table 3).

**Table 3:** Results of three-dimensional model of performance anxiety during baseline and post-golf measurement in control, pre-task, and synchronized music trials.

Parameters	Baseline	Post-test	Wilcoxon test (*P*-value)	% change	Friedman test (Baseline, *P*-value)	Friedman test (Pos *t*-test, *P*-value)	Friedman test (% change, *P*-value)
Cognitive element							
Control	2.15 (1.88, 2.95)	2.00 (1.80, 2.83)	0.146	−3.65 (−13.25, 0.89)			
Pre-task	2.35 (1.88, 2.80)	2.15 (1.88, 2.83)	0.165	0.00 (−13.71, 6.26)	0.419	0.276	0.545
Synchronised	2.60 (1.95, 2.90)	2.25 (1.80, 2.85)	0.727	0.00 (−13.45, 2.92)			
Physiological element						
Control	2.29 (1.82, 3.29)	2.07 (1.57, 3.18)	0.390	0.00 (−10.37, 4.40)			
Pre-task	2.50 (1.89, 3.07)	2.14 (1.57, 3.32)	0.184	−1.92 (−8.94, 1.00)	0.306	0.277	0.012*
Synchronised	2.50 (1.57, 3.00)	2.57 (1.57, 3.43)	0.714	4.58 (0.00, 9.32)			
Perceived element							
Control	2.25 (2.00, 3.06)	2.25 (1.94, 3.25)	0.102	0.00 (−8.48, 11.46)			
Pre-task	2.50 (2.00, 3.25)	2.38 (2.00, 3.50)	0.058	0.00 (−6.67, 1.92)	0.375	0.545	0.845
Synchronised	2.63 (2.00, 3.06)	2.50 (2.00, 3.00)	0.542	0.00 (−7.44, 0.00)			

**Notes.**

Data are presented as median and interquartile range (IQR). An asterisk (*) indicates a significant difference among music conditions (*p* < 0.05). The percentage sign (%) indicates percentage change between pre-test and post-test. Friedman test for inter-condition comparison; Wilcoxon-signed rank test used for pre-test and post-test comparison.

## Discussion

This study aimed to investigate the effects of self-selected music interventions on golf performance and psychophysiological responses after a mental stress test in collegiate golfers. The proposed hypotheses were rejected because there were no significant differences in golf performance, cardiac-related indices, and psychological responses among the experimental conditions (*i.e.,* control, pre-task, and synchronised music trials).

### Effects of music interventions on anxiety level

The results of the present study showed that listening to preferred music (either before or during exercise) following mental stress/fatigue (*i.e.,* after performing the SCWT test) does not reduce anxiety in golfers. These results differ from those of previous sporting studies demonstrating the potential effect of music in counteracting the negative dimensions of mood (*i.e.,* anxiety and tension), and in improving positive attitudes such as motivation and feeling states (*e.g.*, Simpson & Karageorghis, 2006; Stork, Karageorghis & Ginis, 2019). Moreover, the findings did not fit with the neuropsycho”-logical literature showing that music can modify brain function that can provoke positive effects on emotional regulation (Hou et al., 2017), and in reducing fatigue and its related symptoms (Jing & Xudong, 2008). A plausible explanation for the non-positive effects of music on anxiety levels could be related to sleep loss. Indeed, the experimental protocol including the anxiety post-exercise assessment was carried out in the afternoon (*i.e.,* between 13:00 hrs and 15:00 hrs), referred to as the post-lunch dip (Monk, 2005). According to Van Dongen & Dinges (2000), the post-lunch dip is a period of sleepiness that occurs between 13:00 hrs and 16:00 hrs due in part to a slight reduction in core body temperature, which promotes a tendency to sleep (see also Abdessalem et al., 2019; Boukhris et al., 2019). In this context, past scientific works showed that partial sleep deprivation appeared to be more vulnerable to negative mood sensations such as anxiety, fatigue and depression (*e.g.*, Mejri et al., 2016; Andrade et al., 2019; Souissi et al., 2020).

### Effects of music interventions on golf performance

The current study results demonstrated that listening to preferred music following mental stress/fatigue does not affect swing and putting performances in amateur golfers. The findings are not in accordance with earlier research showing the benefits of using music before/during task either on physical performance (*e.g.*, Stork, Karageorghis & Ginis, 2019; Belkhir et al., 2022) or on gross and fine motor skills (*e.g.*, Baghurst et al., 2014). The non-positive effects of music on golf performance could be related to psychological effects. It is well known that psychological state is a determinant element before or during exercise (Enoka & Stuart, 1992; Jones & Killian, 2000). As mentioned in the section above, listening to music following mental fatigue failed to counteract negative mood (*i.e.,* anxiety) in golfers. In this context, Hill & Chtourou (2022) recently demonstrated a causal link between mood state and performance. Another plausible explanation for the non-profit of listening to music following mental stress could be related to the study sample, where most of the participants were males. In this context, it was established that female athletes seemed to derive greater benefit from music during exercise, compared to male athletes (Tounsi et al., 2019). Further studies are needed to test whether our results would also apply if we tack into account such types of individual differences. Otherwise, to further explain the current findings, we could speculate that the non-change in golf performance could be related to the sleep loss during the execution of the experimental protocol (*i.e.,* post-lunch dip period), which led to modest reductions in muscle glycogen, which may signal fatigue to down-regulate muscle activation and prevent glycogen depletion (Romdhani et al., 2019).

### Effects of music interventions on autonomic nervous functions

It was found that listening to preferred music after performing the SCWT test does not affect HR and HRV among collegiate golfers. These results differ from those of previous sporting studies showing the positive effects of music on HR (*e.g.*, Thakare, Mehrotra & Singh, 2017; Patania et al., 2020). Discrepancies between the findings could be related to the potential effect of a musical tempo. In the current study, based on the self-selection mode, we did not control music preference among participants. Indeed, 12 participants choose fast/motivational music with high tempo (>120 bpm), while 8 participants choose slow music with low tempo (<120 bpm) (for more on this point, see Karageorghis et al., 2006). Some earlier research proved that musical tempo may differently influence diverse cardiovascular responses. For example, Copeland & Franks (1991) found that listening to motivational music (>120 bpm) has a more positive effect than listening to slow music (<120 bpm) on HR when performing treadmill endurance exercises. In the same vein, Birnbaum, Boone & Huschle (2009) showed that listening to high musical tempo during steady-state treadmill exercise is more efficient than listening to low musical tempo for cardiovascular responses (*e.g.*, oxygen consumption, stroke volume, frequency of breaths, cardiac output, and systemic vascular resistance). More recently, the relationship between different musical tempos and HR has been examined during endurance (walking for 10 min at 6.5 km/h on a treadmill) and high intensity (80% on 1 repetition maximum) exercises in active adult women (Patania et al., 2020). The authors found showed that HR increased with the increase in musical tempo either during endurance or high-intensity training. Therefore, it would be worthwhile in future studies to compare the effect of listening to different musical tempos following mental fatigue on cardiac-related responses in amateur golfers.

### Limitations and directions for future research

In addition to the above-mentioned limitations (*i.e.,* regarding the individual difference and musical tempo), other questions remain unanswered and require further research. This study is low in ecological validity as we used a computer-based virtual environment (*i.e.,* Golfzon golf simulator) to evaluate golf performance. It is unclear whether the current results would be replicated in a real putting green environment (*e.g.*, Baghurst et al., 2014). Additionally, the intensity of mental stress/fatigue among the participants caused by the SCWT test was not evaluated in this study. As we highlighted above, another limitation is related to the potential effects of music tempo on golf performance and psychophysiological responses in this study. Lastly, this study does not take into account the potential effect of the time of day on performance and mood states. Indeed, the experimental protocol was carried out during the afternoon hours (*i.e.,* between 13:00 hrs and 15:00 hrs). It was established that there is an intraday variation in various exercise performances and mood states (Belkhir et al., 2019; Essid et al., in press). It would be worthwhile to address this issue in future studies.

### Functional implication

Golf is considered a sport based on closed-chain motor control and body coordination. However, psychological and mental aspects are key elements to success in professional competitions. During official tournaments, golfers had tight schedules on consecutive days. The ranking strategy and psychological and physiological strains during long periods of work can cause mental stress that affects golf performance. Music intervention and psychological skills are considered to be optimal strategies to overcome this issue. Despite positive reports of music intervention on psychophysiological modulation and exercise performance in literature, golf competition regulations may not permit the implementation of synchronised music intervention during play. However, golfers can use music tempos to facilitate rhythmic techniques to reduce competition stress occurs. This strategy is supported as an option for improving golf shot accuracy and decreasing the variability of swing performance (Sommer & Rönnqvist, 2009; Sommer, Häger & Rönnqvist, 2014).

## Conclusions

In conclusion, the study found that there are no benefits of pre-task and synchronised music interventions on golf performance, cardiac responses, and reduction of anxiety levels in collegiate golfers after a mental stress task. Although the physical element of the TFAI showed significant differences in the pre- and post-test percentage of physiological elements, no change in cognitive and perceived elements indicated a minimal impact of music intervention on psychological adaptation after the mental stress task when the amateur collegiate golfers practice swing and putting skills in a virtual environment. Future studies should implement game-based environment and competition conditions to simulate real-world situations to explore this relationship.

##  Supplemental Information

10.7717/peerj.13557/supp-1 Supplemental Information 1 Swing performance raw data

10.7717/peerj.13557/supp-2 Supplemental Information 2 Putting STAI-S TFAI raw data

## Additional Information and Declarations

### Data Availability

The following information was supplied regarding data availability: The raw measurements are available in the Supplementary Files.
